# Supplementary material for: Genome-Wide Identification of the Long Noncoding RNAs of Tribolium castaneum in Response to Terpinen-4-ol Fumigation
Source: Insects. 2022 Mar 14;13(3):283. doi: 10.3390/insects13030283 (PMC8951367; doi:10.3390/insects13030283)
Supplement: Supplementary file 1 [file insects-13-00283-s001.zip › Table S3 Statistics of cis- and trans- regulation gene.pdf]

Table S2. Statistics of cis-regulation

Start	Region	TarPairNumber	mRNANumber	lncRNANumber	Flag
0	overlap	2187	1846	1568	Up
1	1-1000	342	333	296	Up
1001	1001-2000	254	248	216	Up
2001	2001-3000	240	239	216	Up
3001	3001-4000	251	248	219	Up
4001	4001-5000	274	273	240	Up
5001	5001-6000	224	222	195	Up
6001	6001-7000	251	247	224	Up
7001	7001-8000	224	218	190	Up
8001	8001-9000	255	250	230	Up
9001	9001-10000	225	222	194	Up
1	1-1000	377	359	297	Down
1001	1001-2000	318	314	273	Down
2001	2001-3000	328	322	264	Down
3001	3001-4000	294	283	243	Down
4001	4001-5000	275	270	223	Down
5001	5001-6000	269	263	222	Down
6001	6001-7000	267	264	229	Down
7001	7001-8000	258	252	211	Down
8001	8001-9000	253	247	209	Down
9001	9001-10000	243	240	210	Down
10001	10001-11000	218	211	187	Down
11001	11001-12000	223	220	200	Down
12001	12001-13000	240	234	197	Down
13001	13001-14000	211	207	181	Down
14001	14001-15000	235	235	191	Down
15001	15001-16000	242	237	213	Down

16001	16001-17000	208	206	171	Down
17001	17001-18000	226	223	191	Down
18001	18001-19000	204	202	166	Down
19001	19001-20000	193	192	165	Down
20001	20001-21000	1	1	1	Down

“Up” indicates lncRNA upstream of mRNA; “Down” indicates lncRNA downstream of mRNA.
