# Supplementary material for: Genome-Wide Identification of the Long Noncoding RNAs of Tribolium castaneum in Response to Terpinen-4-ol Fumigation
Source: Insects. 2022 Mar 14;13(3):283. doi: 10.3390/insects13030283 (PMC8951367; doi:10.3390/insects13030283)
Supplement: Supplementary file 1 [file insects-13-00283-s001.zip › Table S4 mRNA and lncRNA overlap.pdf]

Table S4. status of overlap

Overlap_Class	lncRNA_Num	mRNA_Num	Pair_Num
Lnc-Overlap-mRNA	90	104	110
Lnc-AntiOverlap-mRNA	1070	1279	1444
Lnc-CompleteIn-mRNAExon	5	6	6
Lnc-AntiCompleteIn-mRNAExon	62	74	75
mRNA-CompleteIn-LncExon	10	10	10
mRNA-AntiCompleteIn-LncExon	79	88	89
Lnc-CompleteIn-mRNAIntron	153	172	207
Lnc-AntiCompleteIn-mRNAIntron	143	150	187
mRNA-CompleteIn-LncIntron	10	10	11
mRNA-AntiCompleteIn-LncIntron	35	44	50

Overlap_Class：The category of the overlap; lncRNA_Num：number of lncrnas that overlap with mRNA; mRNA_Num：Number of mRNA overlap with lncRNA; Pair_Num：Lncrna-mrna logarithm of overlap exists.
